# Aging and homeostasis of the hypodermis in the age-related deterioration of skin function

**DOI:** 10.1038/s41419-024-06818-z

**Published:** 2024-06-24

**Authors:** Meiqi Liu, Feng Lu, Jingwei Feng

**Affiliations:** Department of Plastic Surgery, Nanfang Hospital, Southern Medical University, 1838 Guangzhou North Road, Guangzhou, Guangdong 510515 People’s Republic of China

**Keywords:** Chronic inflammation, Experimental models of disease, Stem-cell research, Immunopathogenesis, Biomarkers

## Abstract

Adipose tissues in the hypodermis, the crucial stem cell reservoir in the skin and the endocrine organ for the maintenance of skin homeostasis undergo significant changes during skin aging. Dermal white adipose tissue (dWAT) has recently been recognized as an important organ for both non-metabolic and metabolic health in skin regeneration and rejuvenation. Defective differentiation, adipogenesis, improper adipocytokine production, and immunological dissonance dysfunction in dWAT lead to age-associated clinical changes. Here, we review age-related alterations in dWAT across levels, emphasizing the mechanisms underlying the regulation of aging. We also discuss the pathogenic changes involved in age-related fat dysfunction and the unfavorable consequences of accelerated skin aging, such as chronic inflammaging, immunosenescence, delayed wound healing, and fibrosis. Research has shown that adipose aging is an early initiation event and a potential target for extending longevity. We believe that adipose tissues play an essential role in aging and form a potential therapeutic target for the treatment of age-related skin diseases. Further research is needed to improve our understanding of this phenomenon.

## Facts

- Age-related hypodermal dysfunction and its unfavorable consequences accelerate skin aging-related processes, such as chronic inflammaging, immunosenescence, delayed wound healing, and loss of cell-cell communication.
- Age-related changes in adipose tissues involve the redistribution of deposits and changes in composition in parallel with the functional decline of adipocyte progenitors, defective redifferentiation, accumulation of senescent cells, and improper adipocytokine production.
- As dWAT has been shown to exhibit differentiation and immunoregulatory functions, it is recognized as an important organ for both non-metabolic and metabolic health in skin regeneration and rejuvenation.
- dWAT plays an essential role in aging and is considered an early initiation event in aging and a therapeutic target for the treatment of age-related skin diseases.

## Open questions

- What are the fundamental mechanisms that trigger a cascade of molecular and cellular changes, such as hypodermis-resident cell aging, in response to both endogenous and external stressors?
- How does dWAT interact with hypodermis-resident cells in the maintenance of organismal homeostasis and the regulation of self-renewal, immune potential, and metabolic modulation?
- What type of medical methods aid in interventions with dWAT for delaying aging-related damage in skin homeostasis and function as well as in optimizing the total healthy lifespan of individuals?

## Introduction

The hypodermis, also referred to as subcutaneous tissue, is the innermost layer of the skin in the human body. The hypodermal connective tissue is more important than an element that provides structural support. Its functions include wound healing, preservation of energy homeostasis, body temperature regulation, mechanical force lubrication, and tissue connection [1]. Under the dermal-epidermal junction, there are two types of adipose tissue in the skin subcutaneous adipose tissue and dermal adipose tissue [1].

A decade ago, the terms used to describe the deep reticular layer of the skin were not unified. However, with advanced research on hair follicles and continuous improvements in cell line-tracing technology, the location of the hypodermis between the reticular layer of the dermis and panniculus carnosus in mice has been confirmed [2]. Evidently, the cell renewal frequency, precursor cells, and lineage differentiation in adipose tissue are different from those in fat from other parts of the body, but they are closely related to the growth cycle of dermal hair follicles. These adipocytes and dermal fibroblasts have common precursor cells and are located in the dermis [2]. Therefore, in recent years, scholars have proposed the concept of human dermal white adipose tissue (dWAT) to characterize adipose tissue surrounding the proximal half of the hair follicles. This definition not only indicates the anatomical structure of the skin but also has important implications for adipose research and associated skin diseases.

Extensive advancements in hair follicle research have led researchers to coin the terms “dermal adipose tissue” and “intradermal adipocytes.” From an evolutionary perspective, coordinated changes in dermal fat formation and hair follicles are conducive to improving the adaptability of mammalian skin and are related to seasonal hair growth in animals [3]. In 2013, the adipocyte lineage of dWAT was first found to facilitate acute skin wound healing [3]. At present, dWAT is considered to play an etiological role and is a potential therapeutic target in obesity, lipodystrophy, alopecia, and fibrosis, among other conditions [4].

In contrast to other fat depots, the hypodermis becomes thinner with age [5]. The hypodermis is an important stem cell niche and hormone and adipokine source. It is populated by local and blood-derived immunocytes that contribute to the innate immune system in the skin. Aging in the hypodermis not only contributes to skin thinning and sagging but also considerably jeopardizes the dermal microenvironment and skin function [6].

Multiple pathological mechanisms cause defective redifferentiation, adipogenesis, inflammation, improper adipocytokine production, and immunological dissonance, all of which lead to age-associated dWAT dysfunction [7]. Here, we review age-related alterations in dWAT across levels, emphasizing the underlying mechanisms that regulate aging. We also discuss the pathogenic pathways of age-related fat dysfunction and the unfavorable consequences of accelerated skin aging, such as chronic inflammaging, immunosenescence, delayed wound healing, and loss of cell-cell communication, and summarize our understanding of dWAT as a therapeutic target for the treatment of age-related skin diseases.

## Role of the hypodermis in skin homeostasis

**Fig. 1:**
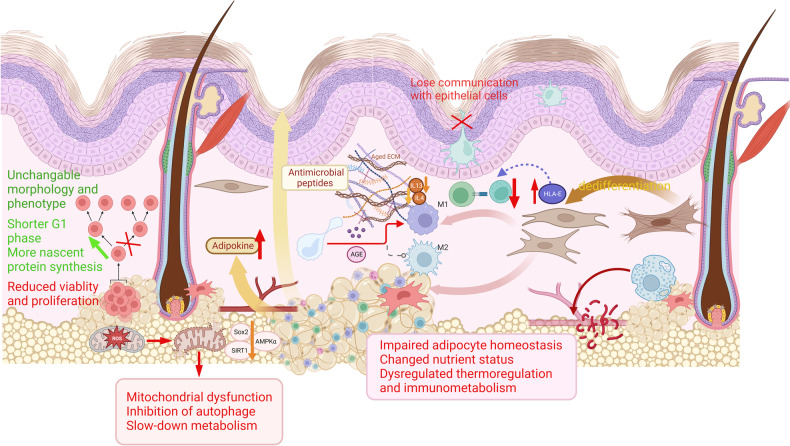
Cutaneous changes in the aged hypodermis. Aging in the hypodermis not only contributes to skin thinning and sagging but also considerably harms the dermal microenvironment and skin function. Aged ASCs exhibit senescent characteristics including reduced viability and proliferation, while the individual morphology and phenotype are independent on donor age. Accumulated oxidative stress during aging reduces the expression of stemness markers and interferes with mitochondrial function and autophagy progression. The immature fibroblast–adipocyte lineage loses its adipogenic-antimicrobial properties with a strengthening of the myofibroblast phenotype and no longer produce antimicrobial peptide in adulthood. Aged immune cells lose their ability to sustain adipose tissue homeostasis and promote healthy aging in various ways. BAT-mediated thermogenesis also declines with age. The aged microenvironment induces an age-dependent increase in the expression of pro-inflammatory mediators and a more proinflammatory M1-like phenotype of macrophages in the hypodermis. Systemic differences in the quantity, intensity, pathway, and signaling mediators of cell–cell communication in young and aged skin have been observed.

As the first line of defense against external pathogens and environmental insults, the cellular components of the skin work in harmony to counter physical and chemical challenges. Hypodermal adipocytes respond to these challenges either alone or in combination with multiple immune cells (Fig. 1).

### Hypodermal stem cell reservoir under senescent phenotype

As people age, the hypodermis tends to thin. The connective tissues joining the dermis to muscles, tissue, and bones also become thinner. In addition, a decrease in energy expenditure with age leads to depot redistribution among visceral, subcutaneous, marrow, intermuscular, and intramuscular adipose tissues, in parallel with the functional decline of adipocyte progenitors, defective redifferentiation, accumulation of senescent cells, and improper adipocytokine production [8, 9]_._

Adipose stem cells (ASCs) from white adipose tissue are thought to constitute the major stem cell population contributing to subcutis regeneration and play an essential role in epithelialization [9–11]. Aged ASCs exhibit senescent characteristics concomitant with reduced viability and proliferation [12]. However, the individual morphology and phenotype of these cells are barely dependent on donor age [7, 12]. Skin preadipocyte proliferation and differentiation capacities are weakened with age [13]. Age is negatively correlated with preadipocyte proliferation in subcutaneous depots, but not in omental depots. Additionally, each fat depot is different, as preadipocyte properties vary according to their localization [14]. Therefore, when the stem cell reservoir for differentiation reduces with aging, the abundance of hypodermal adipose tissue also reduces, with an increase in fibrosis and low adipocyte quantity [8].

### Hypodermal adipose tissue under inflammaging phenotype

**Fig. 2:**
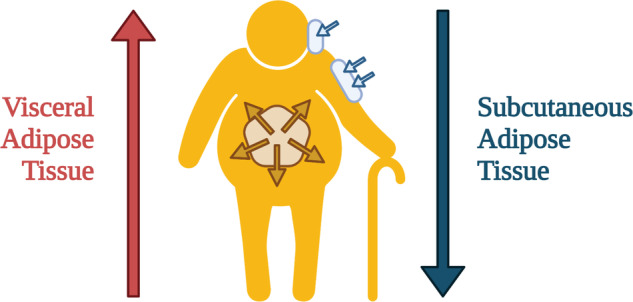
Age-related redistribution of adipose tissues. A decrease in energy expenditure with age leads to depot redistribution among visceral, subcutaneous, and other adipose tissues, in parallel with the functional decline of adipocyte progenitors, defective redifferentiation, accumulation of senescent cells, and improper adipocytokine production. The characteristic manifestation is the increased volume of visceral adipose tissue and the reduced volume of subcutaneous adipose tissue.

Hypodermal AT is a major endocrine organ with strong immunomodulatory properties [15, 16], undergoing changes with age [17]. In healthy individuals, adipose inflammation and metabolic disorders present distinct proaging patterns for stable senescence transformation (Table 1). The mass of subcutaneous adipose tissue is independently reduced with age (Fig. 2). The recently discovered age-dependent regulatory cells (ARCs) play important roles in the age-related reduction of SAT volume [18]. ARCs increase in abundance after middle age and display high levels of pro-inflammatory markers, which usually inhibit the differentiation of new adipocytes and block the expansion of SAT size [18]. Notably, these cells increase in abundance with age and are not affected by high-fat diet consumption in mice. Several researchers consider that the skin may have a distinctive aging pattern owing to its special anatomical location and year-in-year-out environmental exposure [19, 20] (Table 1).

**Table 1:** The aged-related differences in dWAT, sWAT, and vWAT.

	Aged hypodermis white adipose tissue		Aged visceral WAT	Ref.
	dWAT	sWAT	vWAT	
Progenitor clusters	Only one cluster of progenitors is included in a mixed population of SAT and VAT-derived cells.			[54]
Reservoir of ASCs	Less number of effective ASC in WAT			[13, 14]
Bioactivity of the individual stem cells	Unchanged			[15]
Increased burden of senescent cells	+		+!	[17]
SA-β-gal	+++++++		+	[16]
Age-dependent regulatory cells (ARCs)	+		−	[18]
Immune- and inflammation-associated gene expression	+		+!	[21]

+ There is a rise in the amount; −: There is a decrease in the amount; +! It increases more than “+”, but it’s a qualitative result; +++++++ It increases seven times more than “+”.

+ increase in quantity; − decrease in quantity; +! increase greater than “+”, but a qualitative result; +++++++ an increase that is seven times more than that in “+”.

Recent evidence suggests that an increased burden of senescent cells (SCs) in adipose tissue might contribute to the pro-inflammatory phenotype of aging for the effective clearance of cells [21]. Subcutaneous AT showed an excellent effect in the elimination of SCs, alleviating age-related tissue dysfunction and inflammaging. On one hand, the activity of senescent factor SA-β-gal is seven-fold higher in subcutaneous AT than in omental AT in contrast to that of upregulated inflammatory-associated factors such as insulin-like growth factor-binding protein 3 (IGFBP3), plasminogen activator inhibitor 1 (PAI1), C–C motif chemokine ligand 2 (CCL2), and interleukin (IL)-6 [22]. Among them, IL-6 is a pleiotropic cytokine with various physiological and pathophysiological functions. A low or controlled IL-6 release is associated with anti-inflammatory, antioxidant, and pro-myogenic actions, whereas increased systemic levels of IL-6 can induce pro-inflammatory, pro-oxidant and pro-fibrotic responses [23].

As a leading contributor to aging-related health decline, sWAT seems to have a thicker “skin” than vWAT with respect to the downregulating behavior of aging-regulated long non-coding RNA [24, 25]. In pairwise age group comparisons, 1237 genes were identified to be differentially expressed in the skin [26], whereas only a few immune- and inflammation-associated genes were found to be differentially expressed in the brain and blood [26].

### Role of dWAT in skin homeostasis and aging

Skin-associated adipocytes are established in the dermal mesenchyme alongside fibroblast lineages in a manner that is distinct from the development of subcutaneous adipocytes (Fig. 1). This population constitutes a unique adipocyte population in the skin (dWAT) [3, 6].

Considering the heterogeneity of dWAT, the description and definition of stem cell lineages were not uniform in initial studies. Some researchers referred to them as perifollicle stem cells or dermal premature cells [27, 28]. With advancements in research, the cells are now considered a heterogeneous ASC group with characteristics of fibroblasts and macrophages [4, 29]. However, high-throughput sequencing and correlation analyses of the complete lineage composition of these stem cells are yet to be conducted.

dWAT, as an immune reservoir, contains nearly every immune cell type, including mast cells, macrophages, memory T cells, dendritic epidermal T cells, and Langerhans cells [30]. However, even adipocytes are capable of producing adipokines and antimicrobial peptides [31, 32]. Moreover, these immune cells also participate in the non-immune functions of dWAT, such as regulating adipocyte homeostasis and responding to alterations in the nutrient status and body temperature, which indicates the therapeutic potential of the adipose tissue immune system in aging and disease [33, 34].

#### Antimicrobial immunity in dWAT

dWAT is a responsive endocrine organ capable of exerting both local and systemic immune effects [35]. The preadipocyte lineage of dermal fibroblasts in dWAT undergoes rapid in situ proliferation and differentiation and synthesizes the antimicrobial peptide cathelicidin, which can directly combat exogenous infections of the skin [30]. These preadipocytes go down the colocalization of preadipocyte factor 1 (PREF1/DLK1), an early marker of adipogenesis, and cathelicidin, to specifically trigger the production of cathelicidin [31]. Mature fibroblasts respond to infections by inducing the reactive adipogenesis of cathelicidin to stimulate the TGFBR-SMAD2,3 pathway [36, 37]. However, the immature fat and dFB of adipogenic potential progressively lose the ability to produce CAMP in adulthood, along with an increased susceptibility to infection [38].

This age-related process is mediated by the key upstream regulator TGF-β2 in neonatal dFB [38], which causes the immature fibroblast-adipocyte lineage to lose its adipogenic-antimicrobial properties with a strengthening of the myofibroblast phenotype [39, 40]. When treated with PPARγ inhibitors or adipogenic progenitors, cathelicidin production in dWAT and innate immune responses in the skin are also severely compromised [15, 30].

The antibacterial effect of dWAT may be associated with its antioxidant function and maturation process; however, the potential underlying mechanism remains unknown. Recently, retinoids were shown to enhance and sustain the expression of cathelicidin depending on a broadly active transcription factor within the CAMP gene promoter, which results in the induction of hypoxia-inducible factor 1-alpha (HIF1ɑ) [41].

#### Thermo-immune role of dWAT

In mammals, white adipose tissue (WAT) stores energy, whereas brown adipose tissue (BAT) dissipates energy into heat through uncoupling protein 1 (UCP1)-mediated thermogenesis [42]. As humans’ BAT is scarce in the skin, beige adipocytes developed from WAT undergoing the ‘white to brown conversion’ represent a promising strategy to counteract skin dysfunctions [43, 44]. Brown-like, beige, or brite adipocytes in WAT can acquire thermogenic properties and become UCP1-positive following exposure to cold temperature, β3 adrenergic agonist stimulation, or exercise [45, 46].

Beige fat cells develop via the activation of thermogenic genes in mature white adipocytes or through the beige adipogenic differentiation of precursor cells [43, 44, 47]. Activation of adipocyte β3 adrenergic receptors induces pre-existing white adipocytes to undergo dramatic cell programming to adopt the beige phenotype, including massively increased mitochondrial numbers and activity [46, 48]and a profound shift of mitochondrial proteome [49, 50].

BAT-mediated thermogenesis declines with age [51]. Human beige fat activity levels also decline with aging, correlating with a decrease in metabolic rate and an increase in adiposity [52, 53]. Cold-responsive mitochondrial proteolysis is a prerequisite for white-to-beige adipocyte cell fate programming during adipocyte thermogenic remodeling [54]. Augmented mitochondrial protease LONP1 expression raises succinate levels and corrects aging-related impairments in white-to-beige adipocyte conversion and adipocyte thermogenic capacity [54]. Also, the IL-6-knockout (KO) is observed to enhance BAT thermogenesis, but these improvements disappear in elderly KO mice [55].

Cold acclimation suppresses immune response-related pathways in adipose tissues and peripheral blood mononuclear cells (PBMCs) by suppressing the expression of genes involved in antigen recognition and presentation, cytokine signaling, and immune system activation [56]. Emerging evidence shows that when host mice are housed in a warmer environment, they exhibit greater levels of CD8+ helper T cell recruitment and activation, and eventually exhibit increased IFN-γ production and greater expression of the activation markers CD69 and Glut-1 [57]. Subsequently, the recruitment and activation of type 1 immune cells, including CD8+ T cells, TH1 cells, NK cells, and type 1 innate lymphoid cells (ILC1), contribute to the accumulation of proinflammatory macrophages in adipose tissues [58].

#### The homeostatic and reparative role of dWAT-resident immunocytes

Human WAT has 28 distinct cell types, including eight previously uncharacterized immune populations, comprising unique subsets of adipose-resident NK cells, innate lymphoid cells (ILCs), macrophages, and dendritic cells (DCs) [59]. Using cytometry by time-of-flight (CyTOF), researchers have suggested that dendritic cells, particularly CD11b^high^ DC-2, are significantly enriched in visceral WAT, making it more susceptible to obesity-induced inflammation, whereas monocytes are more abundant in subcutaneous WAT [60, 61]. The proportion of immune cells in the visceral and subcutaneous WAT depots was comparable, but the stored APCs had different abundance of cellular subtypes [60].

Aged immune cells lose their ability to sustain adipose tissue homeostasis and promote healthy aging in various ways. By producing the pro-aging factor CCL11 (eotaxin-1), a potent chemoattractant for eosinophils, aged WAT eliminates ATE from adipose tissues and disequilibrates the original positive immune state induced by eosinophil-derived IL-4 [62]. Additionally, predominantly composed of B1-innate B cells, fat-associated lymphoid clusters (FALCs), have recently been found to serve as unique immunological sites that are acutely responsive to pathogens and expand in response to chronic inflammation [63, 64]. Depending on the gradual accumulation of the Nlrp3 inflammasome, non-senescent adipose B cells (AABs) resident in age-induced FALCs expand and continuously express IL-1R, which inhibits IL-1 signaling to reduce AAB proliferation and increase lipolysis as a manifestation of organismal senility [65].

### Adipocyte–myofibroblast transition

Tissue regeneration requires adipocyte-dependent communication for the repair of damaged tissues [66, 67]. Adipocytes residing in the hypodermis undergo lipolysis to efficiently recruit macrophages during inflammation. In response, hypodermal adipocyte-derived cells tend to dedifferentiate and give rise to diverse myofibroblasts in the wound bed in later stages [66]. Interestingly, most dermal myofibroblasts in fibrotic skin arise from adiponectin-positive progenitors residing in the skin. This is indicative of a differentiation process known as adipocyte–myofibroblast transition (AMT) [68](Fig. 1).

In vitro experiments have shown that adipocytes can be induced to form myofibroblasts when transforming growth factor-β (TGF-β) expression and Wnt signaling are activated [69–71]. The effects of TGF-β on adipocyte differentiation and fibrosis are mediated via the inhibition of peroxisome proliferator-activated receptor-γ (PPAR-γ), a key indicator of adipocyte differentiation [72, 73]. When the PPAR-γ gene (PPARG) is mutated, variant PPAR-γ exerts counter-regulatory effects on TGF-β, which leads to anti-fibrotic effects in systemic sclerosis (SSc) [74].

Previous studies have shown that mature dermal adipocytes inhibit the reinitiation of the anagen phase of hair follicle (HF) cycling in depilated adult mice by inducing bone morphogenetic protein (BMP) signaling, which is considered to be initiated in early stages but is lost in later stages [29, 75]. Dermal cells from hair follicles in wounds reprogram myofibroblasts to an adipocyte fate through an HF-independent BMP-ZFP423 pathway, whereas dermal cells without hair follicles do not [76]. BMP2 and BMP4 upregulate adipogenic lineage transcription factors including Zfp423, Crebl2, Stat5b, and klf15 during active cycling [77]. Concurrently, in addition to in situ AMT, wounding was shown to induce the recruitment of Pdgfra^low^–Pdgfrb^high^ myeloid cells, which are a rare subset of adipose precursors contributing to adipocyte regeneration [78].

## Deterioration of stem cell reservoir in the age-related milieu affecting skin homeostasis

Although adipose tissue is primarily composed of adipocytes scaffolded by a web of vasculature, hypodermal WAT also includes preadipocytes, endothelial cells, macrophages, and various other immune cells [79, 80]. The quality and relative quantity of these different cell types, especially preadipocytes, are important for maintaining proper skin function. Committed preadipocytes arise from multipotent, gradually replicating mesenchymal progenitor cells and potentially circulating progenitors [81–83]. Preadipocytes also play critical immunological, proinflammatory, and hemostatic roles and express markers of the monocyte-macrophage lineage [84–86]. Their osteogenic potential is impaired with aging, whereas their adipogenic potential is maintained [87, 88]. A combination of intracellular and extracellular stimuli induces a cascade of transcription factors, including members of the C/EBP family and PPARγ [89–91]. These transcription factors act sequentially to alter the expression of over 2500 genes, which leads to the development of the fat cell phenotype [92, 93].

Preadipocytes from different fat depots exhibit distinct patterns of gene expression, and these depot-specific characteristics inherent to preadipocytes and their corresponding progenitors may contribute to regional functional differences [14, 94, 95]. Compared with that of other progenitors, the abundance of subcutaneous clusters is significantly correlated with factors that enhance a pro-inflammatory state, such as fasting glucose levels [96].

**Fig. 3:**
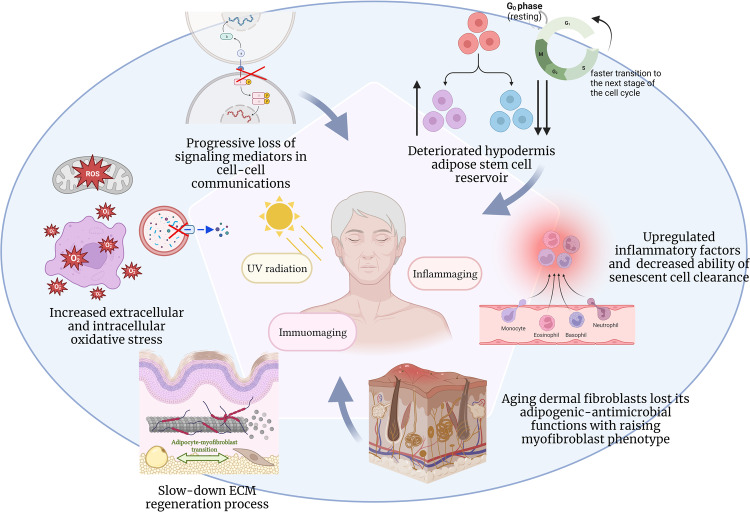
Mechanism of homeostatic and reparative incapacitation in the aged hypodermis. Multiple pathological mechanisms cause defective redifferentiation, adipogenesis, inflammation, improper adipocytokine production, and immunological dissonance, all of which lead to age-associated hypodermis dysfunction. Accumulated extracellular and intracellular oxidative stress is inevitable during aging, which leads to reduced expression of stemness markers and interferes with autophagy progression in human subcutaneous ASCs. Aging affects the structural organization of the dermal and hypodermal ECM. ECM degradation during aging differentially affects immune cell subpopulations and infiltration. Aging dermal fibroblasts lost its adipogenic-antimicrobial functions with raising adipocyte–myofibroblast myofibroblast phenotype. An increased burden of senescent cells in adipose tissue can contribute to the pro-inflammatory phenotype of aging for the effective clearance of cells. Old subcutaneous AT showed a recessive effect in the elimination of SCs, resulting in age-related tissue dysfunction and inflammaging. ASCs tend to adapt to aged, unfavorable environments. During natural chronological aging, ASCs exhibit an increase in nascent protein synthesis and a shortened G1 phase to facilitate a more rapid transition to the subsequent stage of the cell cycle. Systemic differences in the quantity, intensity, pathway, and signaling mediators of cell–cell communication in young and aged skin have also been observed.

Accumulated oxidative stress is inevitable during aging and is accompanied by mitochondrial dysfunction, autophagy inhibition, and the slowing of metabolism [97, 98](Figs. 1, 3)_._ In human subcutaneous ASCs, oxidative stress reduces the expression of stemness markers and interferes with autophagy progression. The expression of proteins such as SRY-related HMG box 2 (Sox2), adenosine 5‘-monophosphate-activated protein kinase α (AMPKα) (a major inducer of autophagy and stress responses) [99], and silent mating type information regulation 2 homolog 1 (SIRT1) (a protective enzyme that plays an important role in cellular metabolism and improves cellular resistance to oxidative stress) [100] and telomerase activity [101] decreased significantly with age. Meanwhile, the level of eukaryotic elongation factor 2 (eEF2) was positively correlated with age [102].

ASCs tend to adapt to aged, unfavorable environments. To meet incremental homeostatic demands during natural chronological aging, ASCs exhibit an increase in nascent protein synthesis and a shortened G1 phase to facilitate a more rapid transition to the subsequent stage of the cell cycle [103]. The differentiation of hypodermal WAT ASCs is also known to be regulated by epigenetic modifications [104], which are less likely to be influenced by ROS, UV radiation, and other genetic senility boosters.

Despite the presence of heterogeneous cell populations, AMT helps replenish damaged adipocytes. In adulthood, relatively limited AMT is observed in subcutaneous and dermal adipose depots. However, under physiological and pathophysiological stress, such as caloric excess, cold exposure, injury, and tumors, mature dermal adipocytes undergo dedifferentiation and redifferentiation via the recruitment of resident adipocyte precursors [105]. Dermal adipocytes are a class of white adipocytes that exhibit excellent deformability. In response to stimuli, adipocytes in dWAT re-express GFP and PDGFRα [106], which are markers expressed only in adipose precursors and preadipocytes but not in mature adipocytes [58]. In parallel with the tumor microenvironment, loose-knit interactions between adipocytes and tumor cells induce AMT and potentially facilitate tumor invasion via extracellular matrix (ECM) remodeling and natural immune stimulation [107, 108]. Adipocytes display myofibroblast- and macrophage-like characteristics and reduce their lipid-storing capacity, which is consistent with previous theories.

## Role of the hypodermis in age-related skin deterioration

### Inflammaging

Aging induces ectopic lipid accumulation, which exacerbates metabolic dysfunction with robust inflammatory and transcriptomic changes [109]. Owing to disrupted homeostasis in continuous fission and fusion cycles and mitophagy, the skin becomes susceptible to a senescence-associated secretory phenotype (SASP) and inflammageing [110, 111]. As aging progresses, a functional loss in the specificity and efficiency of the immune system is induced, which promotes the perpetuation of an ineffective inflammatory provoked state [112, 113]. This constant inflammatory stimulus promotes age-related changes, including the redistribution of adipose tissues and lipoatrophy in the skin [114, 115](Fig. 3).

A systemic decline in autophagic activity with age can subsequently impair homeostasis in skin tissues(Fig. 3), leading to age-related diseases. In contrast to other tissues, aged adipocytes upregulate autophagy based on a decline in Rubicon expression and subsequently exacerbate the excessive autophagic degradation of steroid receptor coactivator-1 (SRC-1) and transcriptional intermediary factor 2 (TIF2) that contribute significantly to adipogenesis [116]. The adiponectin receptor (AR), secreted by subcutaneous adipocytes, attenuates inflammatory factor secretion and apoptosis in aged skin by improving mitochondrial morphology and function [117]. By activating AMP-activated protein kinase (AMPK), AR suppresses dynamin-related protein 1 (Drp1)-mediated excessive mitochondrial division and potentially reduces mitochondrial fragmentation and superoxide synthesis [117].

Although chronic systemic low-grade inflammation may induce metabolic dysfunction, acute localized inflammation can be an adaptive response with positive effects in hypodermal AT remodeling and expansion, thus triggering a response to counteract age-related pro-inflammatory changes [118, 119]. Evidence shows that under adverse circumstances, conserved proteins such as spermatogenesis-associated protein 4 (SPATA4) show tissue-specific functions in promoting preadipocyte differentiation through activation of the extracellular regulated protein kinases (ERK) 1/2 and CCAAT-enhancer-binding proteins β (C/EBPβ) pathways and facilitating adipokine expression in aged mice [120].

### Immunoaging

Similar to that in other organs, both innate and adaptive immune systems in the hypodermal skin undergo functional decline during aging, becoming more fragile and susceptible to infection. This gradual decline, known as immunosenescence [121], may contribute to the incomplete clearance of senescent cells with age [122, 123] (Fig. 3).

The mechanisms underlying immune evasion in senescent skin cells may contribute to their persistence [124]. Senile fibroblasts upregulate HLA-E expression for immune escape, thereby impairing their clearance by NK and CD8+ T cells that express the inhibitory receptor NKG2A [125]. Collectively, senescent fibroblasts produce lysophosphatidylcholine, an SASP factor that can not only stimulate surrounding healthy fibroblasts to release chemokines but also interfere with macrophages with toll-like receptor 2 and 6/CD36 signaling and phagocytic potential, which may encourage immune evasion and low-grade chronic inflammation during long-term skin aging [126].

Concurrently, the aged microenvironment induces an age-dependent increase in the expression of pro-inflammatory mediators and a more proinflammatory M1-like phenotype of macrophages in the hypodermis [127]. This M1-like phenotype downregulates IL-4 and IL-13 and negatively influences the expression of ECM proteins such as collagen type V alpha 1 (Col5a1) and collagen type VI alpha 1 (Col6a1) by fibroblasts, emphasizing the impact on the aged skin phenotype [128]. In addition, advanced glycation end products (AGEs) can induce the differentiation of monocytes into dendritic or macrophage-like cells, leading to the development of a micro-inflammatory environment [129] (Fig. 1).

Aging affects the structural organization of the dermal and hypodermal ECM, in which collagen is sparsely distributed [130, 131] (Fig. 3). The microenvironment of both the dermis and hypodermis is essential for controlling inflammatory attacks and maintaining remission. ECM degradation during aging differentially affects T cell subpopulations and infiltration [132]. By manipulating mechanical changes to produce more youthful ECMs, the infiltration of immune cells, such as T cells, can be improved substantially [133].

### Traumatic injury

Skin wounds in the elderly show significantly delayed healing, similar to chronic diabetic wounds. Aging individuals produce significantly more ROS, which damages critical organelles, reduces cell viability, and delays the healing process [134, 135]. In a senility-impaired milieu, the upregulated miR-21-3p/miR-126-5p/miR-31-5p and downregulated miR-99b/miR-146 axes are considered to play a transcendent role in promoting fibroblast proliferation and migration and regulating innate immune responses and macrophage reprogramming, which interrupts persistent inflammation and substantially delays healing [136–141].

Unconventional inflammatory responses play a critical role in tissue dysfunction and ineffective skin restoration [142]. An expanding body of research suggests that tissue-resident adipocytes and fibroblasts are actively involved in the modulation of inflammation. Although several skin elements, including dWAT and hair follicles, are not renewed in the normal wound bed, in large wounds, macrophages can activate the proliferation of a myofibroblast subset (AP) with adipose-like characteristics that subsequently helps regenerate hair follicles [76, 143]. Age-related variations in the gene expression of extracellular molecules are observed in the transcriptome of myofibroblast populations in young vs. old mice, where myofibroblasts express more metalloproteases with age [143]. This is consistent with the fact that older fibroblasts can degrade the ECM more rapidly than younger fibroblasts in the early stage and hinder recovery [144].

Adipocytes in the dermis can regulate skin wound repair by releasing fatty acids into the wound bed, subsequently facilitating Ly6c^high^ proinflammatory macrophage recruitment and hastening revascularization [66]. Consistent with the changes in AP cells, mature dermal adipocytes dedifferentiate into myofibroblasts, which later migrate and produce extracellular matrix [66]. Recovery can be induced in the skin with radiation-induced wounds by lipid remodeling and the downregulation of lipid metabolites without affecting the volume of the abdominal or visceral adipose tissues [145].

### Fibrosis

After the acute inflammatory and proliferation stages, scar formation is essential for wound healing. Skin lipoatrophy characteristically accompanies dermal fibrosis with the de novo emergence of myofibroblasts to provide sufficient ECM and consolidate mechanical force in SSc, scleroderma, wound repair, hyperplasia scars, or other skin diseases involving skin fibrosis [146–149]. Myofibroblasts, the primary type of skin cells that form scars, appear rapidly in both normal and scar-free Acomys mice but only persist in normal mice after traumatic injury [150]. Many researchers have asserted that ECM-producing myofibroblasts are the primary targets of scar-free healing. Tamoxifen-inducible genetic lineage tracing of mature adipocytes and single-cell RNA sequencing revealed that dermal adipocytes alter their fate and give rise to diverse myofibroblasts to generate extracellular matrix in the wound bed [66].

In the red Duroc porcine model, autologous subcutaneous adipose-derived cell therapy helped modulate IL-6 expression within the scar, with upregulation observed during the early proliferative phase and downregulation in the later scarring phase. This is advantageous for inflammatory cell recruitment to initiate angiogenesis, epithelial reconstruction, and matrix remodeling [151]. Long-term effects, including amelioration of skin hardness, collagen organization, epidermal-dermal junction reconstruction, and hypervascularity, are achieved by regulating the IL-6-trans-signaling-STAT3 pathway [152, 153]. T-lymphocytes, specifically type 1 regulatory (Tr1) T cell subsets, can also promote HA-rich ECM deposition and attenuate the expression of fibrotic components via the polarization of M1 macrophages to M2 macrophages [154]. Dermal fibroblasts co-cultured with Tr1 cells show an increase in HAS and downregulation in connective tissue growth factor (Ctgf) [154].

However, the manner in which these interactions change with age remains unclear. The majority of research on the role of immune cells in the aging hypodermis has been focused on diabetic complications and wound healing. Systemic differences in the quantity, intensity, pathway, and signaling mediators of cell-cell communication in young and aged skin wounds have been observed in these models [155–157]. The greater number of potential signaling interactions in aged skin wounds supports the hypothesis that inefficient healing can result from overactive but misdirected signaling or a lack of proper downstream response [158]. Given that tissue aging is a summation of cumulative conversions in single cells, the approach to pausing skin aging and rejuvenation is significant for understanding aging and the holistic body senile.

## The anti-aging potential of skin cells in dWAT

Adipose-derived stem cell lineages facilitate efficient repair based on their differentiation potential and paracrine functions. The differentiating role of the adipose-derived stem cell lineage in aging has been emphasized in previous articles [18, 159, 160]; here, we focus on its secretion potential. As ADSC-exo is mostly internalized by fibroblasts, its effects on resident immune cells can be indirectly regulated through FBs. Based on high-throughput sequencing results, hypoxic adipose stem cell exosomes (HypADSCs-exo) participate in hypoxia adaptability and accelerate diabetic chronic wound healing by promptly inhibiting inflammation through the phosphoinositide 3-kinase/protein kinase B (PI3K/AKT) signaling pathway [161]. Microvesicles (MVs), which are larger extracellular vesicles, are also capable of advancing aging-induced or inflammation-delayed wound healing. Besides inducing a visible increase in the expression of proliferative markers and growth factors such as myelocytomatosis viral oncogene homolog (c-Myc), matrix metalloproteinase 9 (MMP9), vascular endothelial growth factor receptor (VEGFR), TGF-β, and platelet-derived growth factor A (PDGFA), adipose stem cell-derived microvesicles (ASC-MVs) also stimulate the activation of AKT and ERK signaling pathways in skin cells, which accelerates re-epithelialization, collagen deposition, and neovascularization in vivo and eventually promotes more rapid wound closure [162]. ASC-MVs are perceived as mediators of intercellular communication and can be engulfed by human umbilical vein endothelial cells (HUVECs), HaCAT, and fibroblasts, along with all types of primary skin cells [163].

An antioxidative strategy is beneficial for inhibiting unnecessary inflammation. The overexpression of PRDX4, a member of the antioxidant enzyme family, observed only in adult and aged mice but not in young mice, can help reduce oxidative stress and inflammation by rendering neutrophil recruitment, increasing macrophage infiltration, enhancing angiogenesis, elevating GF levels in vivo, and promoting the proliferation and migration of fibroblasts after injury in vitro [164]. Exosomal PD-L1 can directly bind to PD-1 on the surface of T cells and subsequently suppress T cell activation. This efficiently inhibits excessive and persistent inflammation and ensures the migration of nascent dermal fibroblasts and epidermal cells [165]. MSC-exos have also been demonstrated to possess antioxidant and mitochondrial restoration capacities based on the adaptive regulation of the NRF2 defense system in ameliorating oxidative stress-induced skin injury [166]. The serum levels of TNF-α, IL-1β, and IL-6 and DNA damage are attenuated after the intracutaneous injection of MSC-exos. IL-6 could represent a determinant of the switch from physiologic aging to age-related diseases [167]. Considering that similar manifestations, including ROS accumulation, sustained inflammation, uniform vascularity, and impaired autophagy, are observed in aged skin, these stem cell therapeutic strategies can be applied to hinder skin aging under pathological conditions involving oxidative stress.

The master-subordinate relationship between dWAT hyperplasia and its age-associated reactive immune response remains unclear. However, it is conceivable that mechanisms involving the expansion of the adipose-resident immune system and stronger scavenger cells, such as macrophages, can be beneficial for demic age resistance. Rapamycin, a dietary drug intervention that can significantly extend lifespan, has been proven to induce a 56% increase in CD45+ leukocytes in gonadal white adipose tissue (gWAT), where the majority of these are adipose tissue macrophages (ATMs) responsible for the phagocytosis of apoptotic cells and cellular excreta [168]. Diet-induced or obesity-related genetic dWAT accumulation induces proinflammatory macrophages to secrete overdose TNF and IL-6 via miRNA-containing exosomes [169].

## Summary

With improvements in healthcare and living conditions, the global population currently enjoys an extended lifespan. As individuals age, the capacity of tissues to maintain homeostasis diminishes. This pattern has led to a significant paradigm shift in modern medicine, whereby the focus in the treatment of terminal diseases has shifted to earlier interventions for the prevention and management of aging, along with healthy lifespan optimization. Hypodermis-resident cells play a pivotal role in the maintenance of organismal homeostasis through their ability to differentiate, redifferentiate, and support paracrine functions, immune regulation, and metabolic modulation. These vital functions are considered to be largely regulated by the stem cell lineage in dWAT.

In this review, we highlighted the critical roles of dWAT in aging and hypodermal homeostasis. We discuss the fundamental mechanisms that trigger a cascade of molecular and cellular changes in cutaneous tissue ages in response to both endogenous and external stressors. These modulating factors work simultaneously to alter the biological behavior of hypodermis-resident cells via multiple mechanisms. Although our knowledge of how dWAT contributes to aging and homeostasis maintenance has improved considerably, we still have limited knowledge about how various factors regulate the complex functions of dWAT. Owing to the rapid evolution of single-cell technologies, we can now investigate the aging of adipose tissues by observing several cell types, but the complete picture is yet to be revealed. A compendium of markers that can help study the functions of human dWAT is lacking, especially when presenting key adipocyte techniques associated with lipidomics, metabolomics, and proteomics. Qualitative criteria for the assessment of adipocyte proliferation and apoptosis are also absent.

At present, medical therapy only addresses the clinical consequences of dWAT pathology, without targeting DWAT function, thus impacting the physiology of the skin and associated appendages. It is crucial to use existing medical methods in interventions for delaying aging-induced changes in skin homeostasis and function in dWAT. Since adipose aging interventions can protect against age-related illnesses and systemic aging, additional investigations should be conducted to elucidate the precise processes underlying fat aging and to establish a theoretical framework for antiaging therapy.
